# Causality between alcohol usually taken with meals and Meniere disease: A 2-sample Mendelian randomization study

**DOI:** 10.1097/MD.0000000000037209

**Published:** 2024-02-16

**Authors:** Shihan Liu, Lingli Zhang, Wenlong Luo

**Affiliations:** a Department of Otorhinolaryngology, the Second Affiliated Hospital of Chongqing Medical University, Chongqing, China; b Department of Otorhinolaryngology, Central Hospital Affiliated to Chongqing University of Technology, Chongqing, China.

**Keywords:** alcohol, meals, mendelian randomization, Meniere

## Abstract

The recurrence of Meniere disease (MD) strongly affects patient quality of life. Identifying the risk factors for MD is highly important for its prevention and treatment. Previous studies have suggested that alcohol intake may play a role in the development of MD. However, recent studies have shown that the causal relationship between alcohol consumption and MD remains controversial. In this paper, the Mendelian randomization (MR) method was used to determine the causal relationship between alcohol consumption usually consumed with meals and MD, with the aim of providing suggestions for alcohol intake management in individuals with MD and helping in the prevention and treatment of MD. Two-sample MR was used to investigate the causal relationship between alcohol usually taken with meals and MD. We used a dataset from a publicly available large-scale genome-wide association study (GWAS). Inverse variance weighting (IVW), MR–Egger, simple weighting, weighted weighting and the weighted median method were used for analysis. The final results showed that IVW (OR = 0.991, 95% CI: 0.983–0.998, *P* = .016) results suggested that there was statistical significance, but MR–Egger (OR = 0.978, 95% CI: 0.886–1.080, *P* = .679), weighted median methods (OR = 0.994, 95% CI: 0.985–1.004, *P* = .307) and Simple mode (OR = 0.995, 95% CI: 0.980–1.010, *P* = .566), Weighted mode (OR = 0.995, 95% CI: 0.981–1.010, *P* = .557) found no significant causal relationship. The results suggest that alcohol usually taken with meals may be negatively correlated with MD.

## 1. Introduction

Meniere disease (MD) is currently recognized as a common pathway of symptoms characterized by rotary vertigo, sensorineural hearing loss, tinnitus, and a sense of fullness or pressure in the ear.^[1]^ The reported incidence of MD varies widely globally, with Meniere prevalence being 190/100,000 in the United States.^[2]^ Patients with long-term MD recurrence and vertigo and tinnitus are prone to further depression,^[3]^ cognitive function changes^[4]^ and other emotions, which greatly affects the quality of life of patients and damages their physical and mental health.^[5]^ Therefore, further identification of the risk factors for MD is essential for its prevention and treatment.

MD is a chronic progressive disease, and lifestyle modification is crucial for its prevention. The association between alcohol consumption and MD, a common lifestyle trait, is not clear. Alcohol consumption is currently recognized as a risk factor for several chronic diseases, such as cardiovascular disease^[6]^ and dementia.^[7]^ Alcohol consumption is also considered a risk factor for MD, and limiting alcohol intake in dietary interventions for MD is thought to play a role in preventing the recurrence of MD.^[8]^ According to the guidelines for MD, the first step in the treatment of MD is to avoid caffeine and alcohol.^[1]^ However, in previous reviews, insufficient randomized controlled trials were found to provide evidence to support or refute the idea that MD should limit alcohol intake.^[9,10]^ In addition, contrary to conventional advice, some clinical studies have shown that alcohol consumption may be negatively associated with MD,^[11–14]^ but causal inference has remained unclear. Several studies have shown that average alcohol intake cannot fully explain the causal relationship between alcohol consumption and disease incidence, and different drinking patterns may have different effects on the occurrence and development of disease.^[15]^ To clarify the association between alcohol intake methods and MD for guiding the adjustment of MD lifestyles, in this study we aimed to determine the causal relationship between alcohol usually taken with meals and MD through Mendelian randomization (MR).

MR uses genetic variation as an instrumental variable (IV) to identify causal relationships between 2 traits through analysis of publicly available datasets.^[16]^ This method avoids the limitations of traditional observational studies as much as possible and eliminates the interference of confounding factors on the study results. MR research for various confounding factors cannot test this hypothesis, and this has been confirmed in previous studies.^[17]^ In this study, we analyzed the correlation between alcohol usually taken with meals and MD through a 2-sample MR method.

## 2. Methods

### 2.1. Study design and data sources

**Figure 1.**
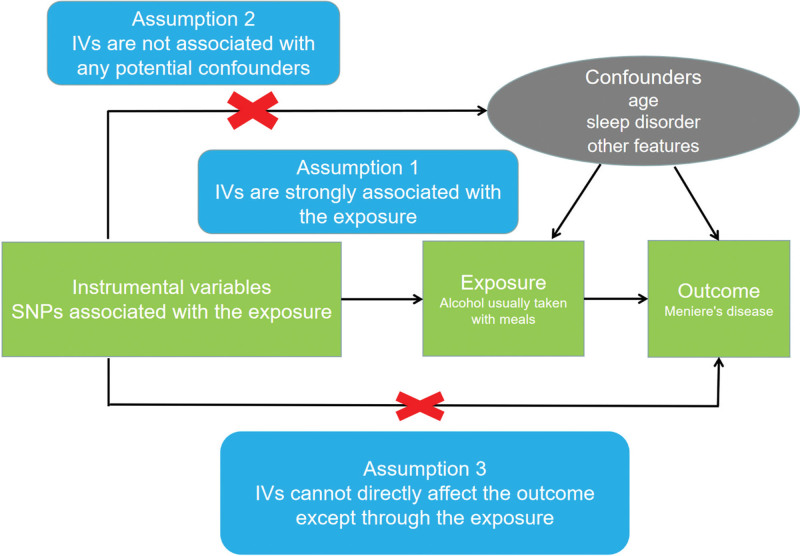
Flow chart of the Mendelian randomization study of alcohol usually taken with meals and for patients with Meniere disease.

The data used in this study were all from publicly available genome-wide association studies (GWASs) and provided ethical approval and informed consent. The flow chart is as follows (Fig. 1). In this study, we conducted 2-sample MR analysis by using GWAS data from multiple public databases. In the MR analysis, we followed 3 criteria (*P* < 5 × 10 − 8 and F > 10^[18]^): genetic instrumental variables strongly correlated with exposure; genetic instrumental variables correlated with outcomes only through exposure; and genetic instrumental variables independent of other confounding factors.

We selected GWAS data from individuals of European descent from IEU GWAS database (https://gwas.mrcieu.ac.uk/) and determined the alcohol usually taken with meals (ncase = 116941, ncontrol = 55,513, number of SNPs = 10,894,596) and MD datasets (ncase = 1321, ncontrol = 461,612, number of SNPs = 9851,867). The dataset of alcohol usually taken with meals comes from the Neale Laboratory Analysis of UK Biobank Phenotypes, round 1. The dataset for the MDs comes from the IEU analysis of UK Biobank phenotypes.

### 2.2. Selection of genetic instruments

By using a 2-sample MR package in R language, we first screened single nucleotide polymorphisms (SNPs) with genome-wide significance (*P* < 5 × 10^−8^) and without linkage imbalance (*r*^2^ < 0.001 and 10,000 KB) in alcohol usually taken with meals dataset, and calculated the F-statistic. IVs with F < 10 are deleted as weak tool variables. Second, if the SNP representing the result was not available in the GWAS data, we directly deleted the SNP. Then, we inferred the positive chain alleles using the allelic frequencies of the palindromes. We scrutinized these SNPs using PhenoScanner to address the phenotypes associated with each SNP and to minimize confounders, such as age, sleep disorders, and other features associated with MD. The SNPs associated with confounders and outcomes were then manually screened and removed. Finally, we analyzed the relevant SNPs.

### 2.3. Statistical analysis

We combined MR estimates by using inverse variance weighting (IVW) as the primary method. MR–Egger,^[19]^ simple mode, weighted mode and weighted median methods^[20]^ were used for sensitivity analyses. The directions of causal associations expressed by the 5 methods are consistent, indicating that the results are significant, and a *P* value <.05 indicates that the results are significant. IVW has the highest statistical efficiency among the 5 methods and is considered the main result of this study. MR–Egger is used to display a nonzero intercept value, mainly to check for horizontal pleiotropy. The causal relationship between alcohol usually taken with meals and MD was binary. We assessed causality using the odds ratio (OR) and 95% confidence interval (95% CI) to determine significance.

Next, we executed the MR–Egger method to obtain intercept values to evaluate horizontal pleiotropy. The Q statistic from Cochran IVW was then utilized to investigate the impact of heterogeneity. Finally, we conducted leave-one-out analyses to explore whether individual SNPs produce biases that influence the overall causal effect.

MR analysis was performed in R version 3.4.2 Computing Environment (http://www.r-project.org) using the TwoSampleMR package.^[21]^ The TwoSampleMR package can analyze the degree of association between exposure and the resulting datasets.

### 2.4. Statistical power

We used the online tool mRND to calculate the statistical power of this MR analysis, which can be used to verify the reliability of the results.^[22]^

## 3. Results

**Figure 2.**
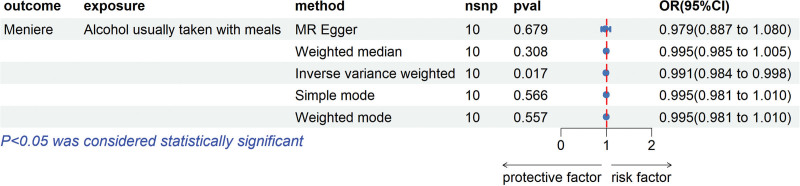
Summary view of the MR results. Summary of MR analysis results derived from the inverse variance weighted, MR–Egger, simple mode, weighted median methods and weighted mode methods. Alcohol usually taken with meals was used as the exposure, and MD was used as the outcome. MD = Meniere disease, MR = Mendelian randomization.

For the dataset of alcohol usually taken with meals, we screened a total of 15 SNPs related to alcohol usually taken with meals (*P* < 5 × 10-8). After matching with the MD GWAS dataset, we removed the SNPs that had no results and ensured that all F-statistics were >10 (range 30–43). Finally, we obtained 10 relevant SNPs for MR analysis. The final results showed that IVW (OR = 0.991, 95% CI: 0.983–0.998, *P* = .016) results suggested that there was significant statistical significance, but MR–Egger (OR = 0.978, 95% CI: 0.886–1.080, *P* = .679), weighted median methods (OR = 0.994, 95% CI: 0.985–1.004, *P* = .307) and Simple mode (OR = 0.995, 95% CI: 0.980–1.010, *P* = .566), Weighted mode (OR = 0.995, 95% CI: 0.981–1.010, *P* = .557) did not find a significant causal relationship between alcohol usually taken with meals and MD (Fig. 2).

**Figure 3.**
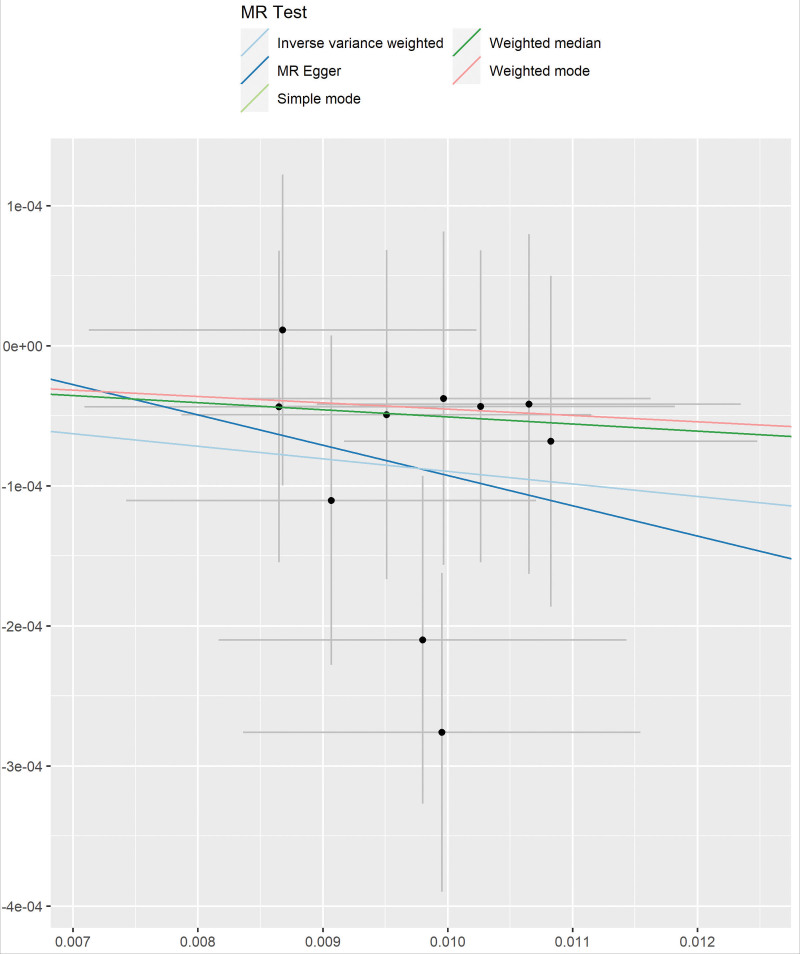
MR test scatter plots of the 5 methods. The x-axis is the SNP effect on alcohol usually taken with meals. The y-axis is the SNP effect on MD. MD = Meniere disease, MR = Mendelian randomization, SNPs = single nucleotide polymorphisms.

**Figure 4.**
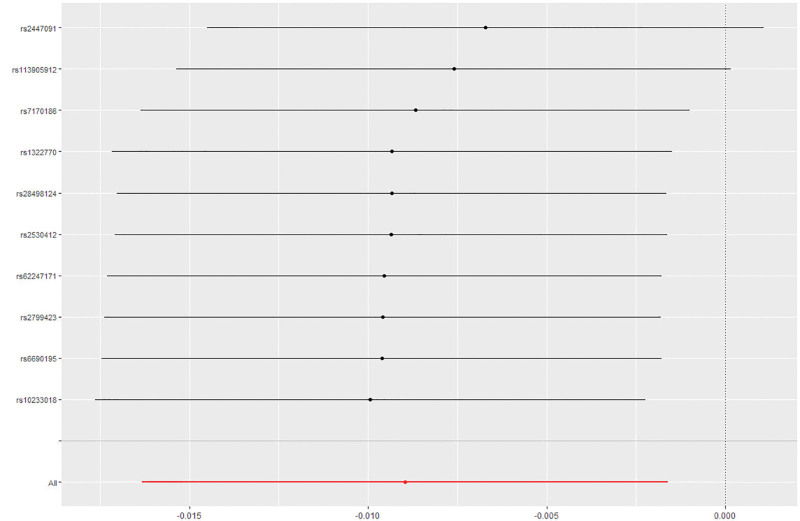
A leave-one-out analysis of the estimations for alcohol usually taken with meals and MD. MD = Meniere disease.

**Figure 5.**
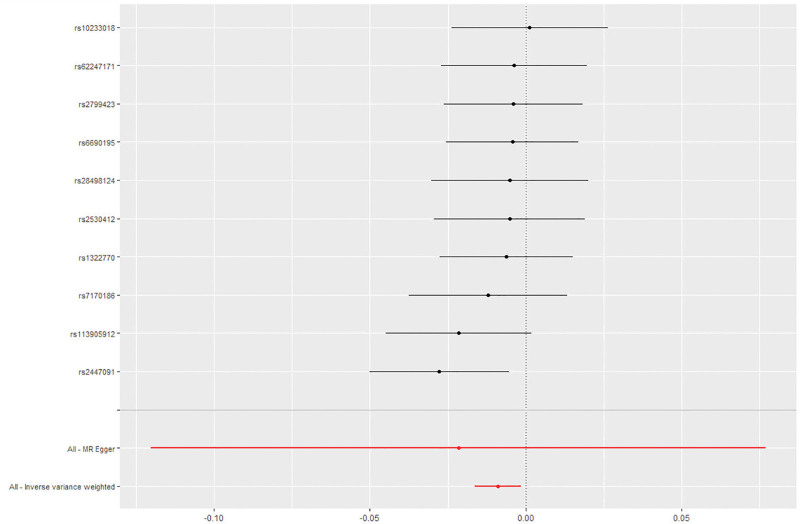
Forest plot of the MR sensitivity analysis. IVM methods showed that MR effect sizes smaller than 0 indicated that alcohol usually taken with meals had a causal effect on MD. MD = Meniere disease, MR = Mendelian randomization.

No horizontal pleiotropy was observed by the MR–Egger intercept test (*P* > .05) (Fig. 3). The heterogeneous results of the MR–Egger assessment (Cochran Q = 8; *P* = .730) and IVW (Cochran Q = 9; *P* = .806) indicated that there was no heterogeneity among the selected IVs. A leave-one-out analysis of the estimations for alcohol usually taken with meals and MD suggested that individual SNPs cannot affect overall causality (Figs. 4–5). The results suggest that alcohol usually taken with meals may be negatively correlated with MD. The statistical power of this MR study (5%) was calculated to be <80%.

## 4. Discussion

In previous studies, there was no clear cause-and-effect relationship between alcohol consumption and MD. In this study, through the analysis of 2-sample MR data, the results showed that alcohol usually taken with meals had a significant causal relationship with MD, and alcohol usually taken with meals had a negative correlation with MD, which was inconsistent with the recommendations for dietary treatment of MD. Moreover, sensitivity analysis and horizontal pleiotropy analyses validated the robustness of the main results.

Since alcohol consumption is considered a risk factor for inner ear diseases, patients with cochleo-derived diseases are often advised to limit alcohol consumption.^[23]^ Current treatment recommendations for MD suggest that patients avoid caffeine and alcohol and maintain a low-salt diet.^[1]^ In contrast to treatment recommendations, some studies suggest that there is no significant association between alcohol intake and MD or that alcohol intake in men may delay the development of MD according to sex.^[12,14]^ Our results also do not support treatment recommendations for MD, and our results suggest that alcohol usually taken with meals may inhibit the occurrence and development of MD. Min Hee Kim et al reported that a decrease in drinking frequency increases the incidence of MD,^[13]^ and Ines Sanchez-Sellero et al suggested that alcohol intake may delay the onset of MD. These findings support the results of our study.^[11]^ Unlike other studies, we did not examine the association between alcohol intake and MD; rather, we examined the causal association between alcohol intake and MD by drinking alcohol with or without meals.

The mechanism underlying the effects of alcohol and MD is unclear, but it is thought that this effect may be related to the use of vasopressin. The reduction in plasma vasopressin levels after endolymphatic sac drainage in patients with MD suggests that vasopressin may have an effect on patients with MD.^[24]^ When alcohol is consumed, the dose of vasopressin decreases,^[25]^ and vasopressin can induce disturbances in patients with endolymphatic hydrops.^[26]^ Disorder of the endolymphatic fluid is considered the signature feature of MD.^[27]^ Therefore, a decrease in the dose of vasopressin during drinking may delay the development of MD. The protective effect of alcohol consumption with meals on MD patients may be attributable to the fact that people who drank alcohol moderately with meals felt happier and calmer after meals and had lower fatigue scores after drinking alcohol with meals than did those who did not drink alcohol.^[28]^ These emotional changes help to stabilize the mood of MD patients, reduce the incidence of anxiety or depression, and inhibit the occurrence and development of further symptoms such as vertigo.^[29]^

No MR study to date has analyzed the causal relationship between alcohol usually taken with meals and MD. The advantage of our study is that the use of large-scale GWAS datasets for MR analysis not only reduces the occurrence of confounding in traditional observational studies but also avoids the bias that may be caused by reverse causality and measurement errors. Moreover, the GWAS data in this study were all from European populations, reducing the influence of race/ethnicity on the results, which makes potential confounding factors more likely to be reduced.^[30]^ However, there are potential limitations to MR analysis. First, as a data simulation analysis, although we did not find that horizontal pleiotropy occurred, we cannot completely rule out the possibility that horizontal pleiotropy affected our results. Second, our MR analysis considered only the relationship between alcohol usually taken with meals and MD, and did not further analyze the time and amount of alcohol consumption; these factors must be further improved upon in future studies. In addition, the data used in our study were obtained from European populations, and further tests in populations from other regions may be needed to verify the generalizability of our findings.^[31]^ Finally, our OR was too close to 1, and the power value was too small; therefore, additional samples may be needed in the future to analyze the association between alcohol usually taken with meals and MD. The results of this study must be viewed with caution in future analyses.

In conclusion, this 2-sample MR analysis revealed that alcohol usually taken with meals may alleviate the occurrence and development of MD. Long-term and repeated vertigo symptoms in MD patients can lead to excessive anxiety,^[32]^ major depression^[33]^ and other mental diseases, which seriously affect quality of life and physical and mental health. Therefore, identifying the risk factors or prevention factors for MD is essential for improving the quality of life of patients and preventing further occurrence and development of other physical and mental diseases.

## Acknowledgments

We thank the UK Biobank and IEU Database for providing GWAS data.

## Author contributions

**Conceptualization:** Shihan Liu, Lingli Zhang.

**Data curation:** Shihan Liu, Lingli Zhang.

**Formal analysis:** Shihan Liu, Lingli Zhang, Wenlong Luo.

**Funding acquisition:** Lingli Zhang.

**Investigation:** Lingli Zhang.

**Methodology:** Lingli Zhang.

**Project administration:** Lingli Zhang, Wenlong Luo.

**Resources:** Lingli Zhang.

**Software:** Shihan Liu, Lingli Zhang.

**Supervision:** Lingli Zhang, Wenlong Luo.

**Visualization:** Shihan Liu, Lingli Zhang.

**Writing – original draft:** Shihan Liu, Lingli Zhang.

**Writing – review & editing:** Wenlong Luo.
